# Salivary, serological, and cellular immune response to the CoronaVac vaccine in health care workers with or without previous COVID-19

**DOI:** 10.1038/s41598-022-14283-x

**Published:** 2022-06-16

**Authors:** Marina Mazzilli Ortega, Laís Teodoro da Silva, Érika Donizetti Candido, Yingying Zheng, Bruna Tiaki Tiyo, Arthur Eduardo Fernandes Ferreira, Simone Corrêa-Silva, Guilherme Pereira Scagion, Fabyano Bruno Leal, Vanessa Nascimento Chalup, Camila Araújo Valério, Gabriela Justamante Händel Schmitz, Carina Ceneviva, Aline Pivetta Corá, Alexandre de Almeida, Edison Luiz Durigon, Danielle Bruna Leal Oliveira, Patricia Palmeira, Alberto José da Silva Duarte, Magda Carneiro-Sampaio, Telma Miyuki Oshiro

**Affiliations:** 1 Laboratorio de Investigacao Medica em Dermatologia e Imunodeficiencias (LIM 56), Faculdade de Medicina, Hospital das Clinicas HCFMUSP, Universidade de Sao Paulo, Av. Dr. Eneas Carvalho de Aguiar, 470, Predio 2, 3º andar, Cerqueira Cesar, São Paulo, SP CEP: 05403-000 Brazil; 2 Laboratorio de Virologia Clinica e Molecular do Instituto de Ciencias Biomedicas da Universidade de São Paulo, São Paulo, SP Brazil; 3 Departamento de Pediatria, Faculdade de Medicina, Universidade de Sao Paulo, São Paulo, SP Brazil; 4 Laboratorio de Pediatria Clinica (LIM 36), Departamento de Pediatria, Faculdade de Medicina, Hospital das Clinicas HCFMUSP, Universidade de Sao Paulo, São Paulo, SP Brazil; 5 Divisao de Laboratorio Central, Faculdade de Medicina, Hospital das Clinicas HCFMUSP, Universidade de Sao Paulo, São Paulo, SP Brazil; 6 Plataforma Científica Paster-USP, Universidade de Sao Paulo, São Paulo, SP Brazil; 7 Hospital Israelita Albert Einstein, São Paulo, SP Brazil

**Keywords:** Infectious diseases, Adaptive immunity

## Abstract

We investigated the anti-SARS-CoV-2 post-vaccine response through serum and salivary antibodies, serum antibody neutralizing activity and cellular immune response in samples from health care workers who were immunized with two doses of an inactivated virus-based vaccine (CoronaVac) who had or did not have COVID-19 previously. IgA and IgG antibodies directed at the spike protein were analysed in samples of saliva and/or serum by ELISA and/or chemiluminescence assays; the neutralizing activity of serum antibodies against reference strain B, Gamma and Delta SARS-CoV-2 variants were evaluated using a virus neutralization test and SARS-CoV-2 reactive interferon-gamma T-cell were analysed by flow cytometry. CoronaVac was able to induce serum and salivary IgG anti-spike antibodies and IFN-γ producing T cells in most individuals who had recovered from COVID-19 and/or were vaccinated. Virus neutralizing activity was observed against the ancestral strain, with a reduced response against the variants. Vaccinated individuals who had previous COVID-19 presented higher responses than vaccinated individuals for all variables analysed. Our study provides evidence that the CoronaVac vaccine was able to induce the production of specific serum and saliva antibodies, serum virus neutralizing activity and cellular immune response, which were increased in previously COVID-19-infected individuals compared to uninfected individuals.

## Introduction

Coronavirus disease 2019 (COVID-19) caused by severe acute respiratory syndrome coronavirus 2 (SARS-CoV-2) was first described in the city of Wuhan, Hubei Province, China, from which it spread widely, gaining pandemic status and changing the global lifestyle^1^. The genomic organization of SARS-CoV-2 is composed of an enveloped single positive-stranded RNA genome that encodes four structural proteins: a spike (composed of S1 and S2 portions), membrane (M), envelope (E) and nucleocapsid (N)^2,3^.

As SARS-CoV-2 continues to circulate in the human population, potentially more infectious and transmissible variants may emerge that harbour mutations in the viral S protein, which is considered the major target of neutralizing antibodies (reviewed by Hirabara et al.)^4^. In fact, some mutants have been a matter of concern, such as the Alpha (B.1.1.7), Beta (B.1.351), Gamma (P.1), Delta (B.1.617.2) and the more recent Omicron (B.1.1.529) variants ^5^. These mutants can rapidly become the dominant circulating virus strains, presenting the potential to spread globally.

The course of SARS-CoV-2 infection depends on several factors, including patient age, the presence of comorbidities and immune responses. Likewise, in most recovered patients, the presence of SARS-CoV-2-specific antibody titres and virus-specific memory CD4+ and CD8+ T cells are observed^6–10^, which can indicate the development of protective immunity.

Since December 2020, several COVID-19 vaccines have been approved by the World Health Organization (WHO) Emergency Use Listing Procedure^11^, and they are currently being administered worldwide. Most of these vaccines target the SARS-CoV-2 S protein using viral vectors (AstraZeneca/Oxford and Janssen Ad26.COV2. S/Johnson & Johnson) or mRNA (Moderna and Pfizer/BioNTech) or they target the entire inactivated virus (CoronaVac/Sinovac and Sinopharm/China National Pharmaceutical Group). Importantly, this first vaccine generation was developed based on the ancestral strain, without mutations, raising serious concerns about neutralizing antibody responses elicited by these strains.

CoronaVac is an anti-COVID-19 vaccine based on the inactivated virus that was the first to be used on a large scale in Brazil. Unlike other vaccines in use, based on the spike protein, CoronaVac provides the whole virus antigenic repertoire for the immune system, which can influence the post-vaccine immune response profile.

Vaccine immune response evaluation is generally performed by monitoring specific antibody titres in the blood; however, little is known about the immune response at the site of infection, such as the oral mucosa. In addition, it is known that there is a decline in antibody levels months after infection or immunization^12–14^, but sustained T-cell immunity has been shown to be related, despite a decline in the antibody response^15,16^, suggesting that both humoral and cellular immunity are required for protection.

Measurements of antibody levels are accessible and practical, unlike evaluating cellular response profiles, which is complex and requires cell culture assays. However, cellular testing allows for the stimulation of cells, enabling access to a cellular memory profile, which can often be hidden until immune system stimulation. In this context, further studies are needed to clarify the contribution of both antibodies and immune cells in protecting against infection. Additionally, evaluating the performance of serum samples regarding their ability to neutralize viral activity against SARS-CoV-2 variants, through neutralizing antibodies is another important point to consider, since reports of reduced protection against these variants have been described^17^.

In this context, few studies have been focused on analysing different immune compartments in individuals immunized with inactivated virus-based vaccines with or without previous COVID-19. Here, we evaluated samples from volunteers who recovered from COVID-19 and/or were vaccinated with CoronaVac for the presence of serum and salivary anti-SARS-CoV-2 antibodies, a serum neutralizing ability against SARS-CoV-2 variants and specific T-cell responses.

## Results

### Volunteer characteristics

Samples from 115 donors were included in the study. Their ages ranged from 20 to 48 years old, with the uninfected and unvaccinated (UI/UV) donors being younger than the volunteers from other groups (*p* < 0.01). Among those recovered from COVID-19 and vaccinated individuals (REC/VAC), all of them were female (*p* < 0.05), which can be explained because this group is part of a children’s health hospital staff, in which most of the employees are women. REC/VAC individuals were diagnosed by positive RT-qPCR for SARS-CoV-2 using a nasal/oral swab sample at least 30 days before inclusion in the study or, in some cases, by confirmation after the absence of symptoms through serological detection of IgG anti-SARS-CoV-2 antibody. The time from the second vaccine dose to study entry was longer in the REC/VAC group than in the VAC group (*p < 0.05). Lastly, the time elapsed between infection and study entry ranged from 1 to 12 months, and the clinical form varied from asymptomatic to mild. The characteristics of the volunteers are described in Table 1.

**Table 1:** Demographic and clinical characteristics of the participants of this study.

Group	Age (years)	Sex	Time from COVID diagnosis to study entry (months)	Time from 2nd dose to study entry (days)
VAC (n = 80)	35 (31–48)	F (91.4%): M (8.5%)	N/A	66 (63–71)^c^
REC/VAC (n = 22)	33.5 (28.5–35.7)	F (100%): M (0%)^b^	6.5 (4–10.7)	68 (66–73.5)
UI/UV (n = 13)	23 (23–31)^a^	F (60.8%): M (39.1%)	N/A	N/A

The median (25% and 75% IQ, interquartile values) for the age, time from COVID diagnosis to study entry, and time from second dose of CoronaVac to study entry are shown.

*REC/VAC* recovered and vaccinated, *VAC* vaccinated, *UI/UV* uninfected/unvaccinated individuals, *F* female, *M* male, *N/A* non-applicable.

^a^Statistical significance was observed between the ages of UI/UV compared to VAC (***p < 0.001) and REC/VAC individuals (**p < 0.01).

^b^Statistical significance was observed between the sexes of REC/VAC and UI/UV individuals (*p < 0.05). One-way ANOVA and the nonparametric Kruskal–Wallis test were used to compare the three study groups.

^c^Statistical significance was observed between the time of the second dose and study entry in REC/VAC and VAC (*p < 0.05). T tests and nonparametric Mann–Whitney tests were used to compare these vaccinated groups.

### Serum levels of IgG anti-SARS-CoV-2 antibodies

**Figure 1:**
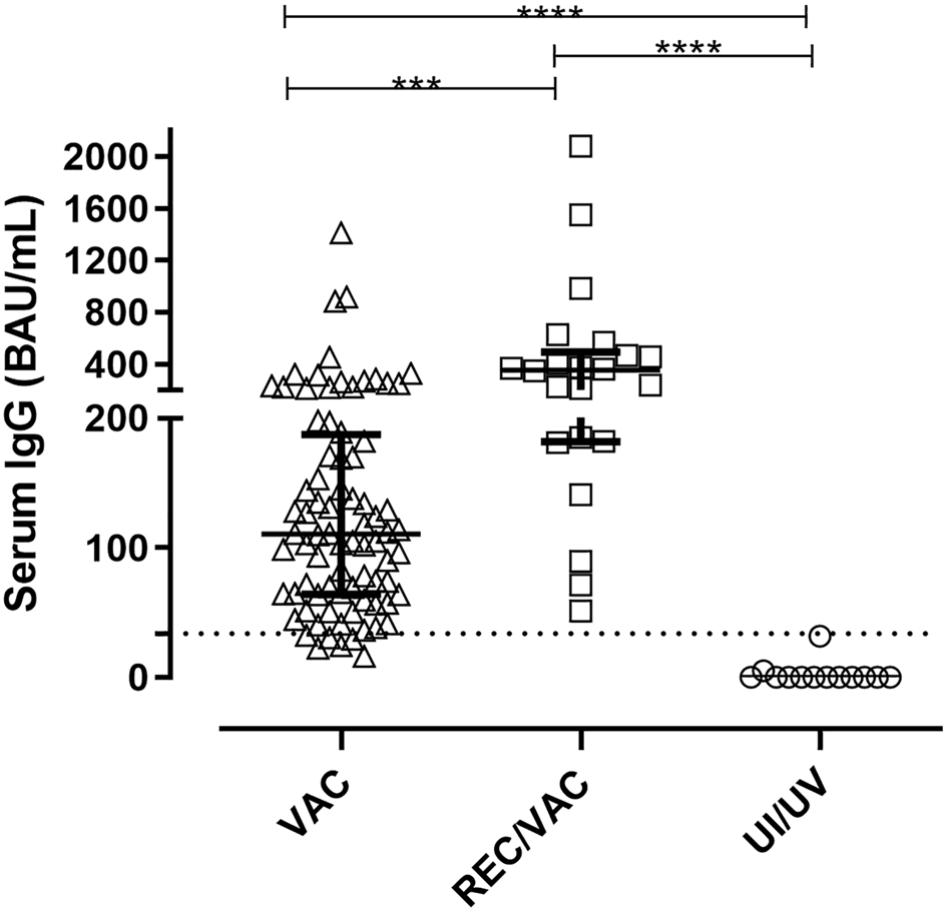
Serum IgG antibodies against SARS-CoV-2 spike protein. Serum from COVID-19 vaccinated (VAC) (n = 80; triangles) patients, those who were recovered from COVID-19 and vaccinated (REC/VAC) (n = 22; squares) and negative control (UI/UV) individuals (n = 13; circles) were analysed to measure the IgG antibodies anti-SARS-CoV-2 S1 and S2 proteins. Scatter plots show lines at the median with interquartile ranges. The dashed line represents the cut-off value for the test (33.8 BAU/mL). One-way ANOVA and the nonparametric Kruskal–Wallis test were used to compare the study groups. Asterisks denote statistical significance between the groups (***p < 0.001; ****p < 0.0001).

The serum samples were analysed for the presence of IgG antibodies directed to the SARS-CoV-2 trimeric spike glycoprotein (Fig. 1). CoronaVac was able to induce specific IgG antibodies in most VAC group volunteers (> 92%). The REC/VAC group presented positive responses for all samples (100%) and at levels significantly higher than those of the VAC group.

In contrast to groups who were vaccinated (VAC and REC/VAC), IgG anti-SARS-CoV-2 spike-specific antibodies were not detectable in samples from the UI/UV group. Statistical significance was observed in this analysis (***p < 0.001).

### Neutralization of SARS-CoV-2 lineages by serum samples

**Figure 2:**
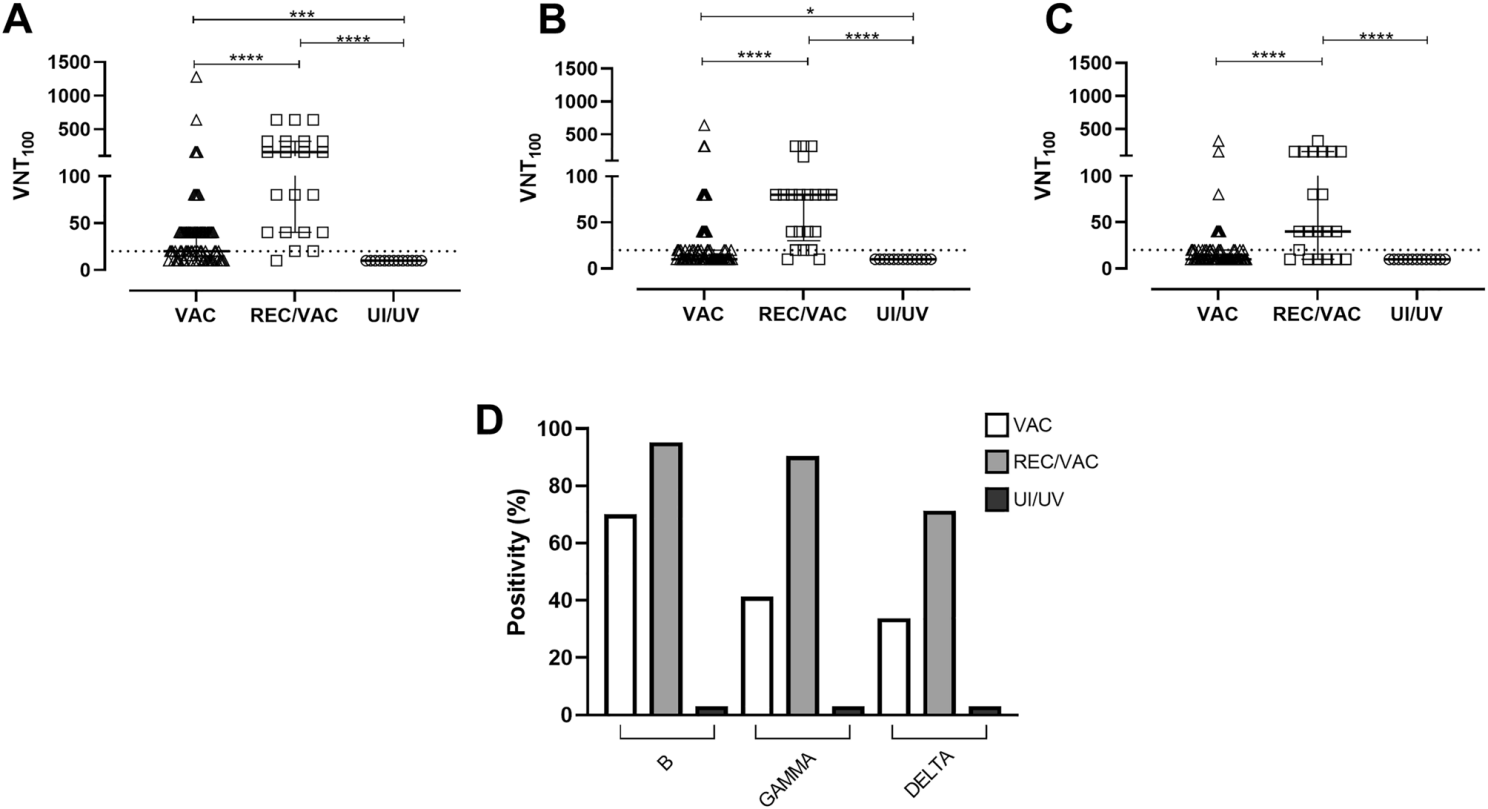
Neutralization of SARS CoV-2 lineages B, Gamma and Delta with serum from previously infected and/or vaccinated individuals, according to their VNT100. Serum obtained from COVID-19 vaccinated individuals (VAC) (n = 80; triangles), those recovered from COVID-19 and vaccinated (REC/VAC) (n = 21; squares) and negative control (UI/UV) individuals (n = 13; circles) were analysed to determine the virus neutralization titre (VNT_100_) to the reference SARS-CoV-2 lineage B (**A**); Gamma (**B**) and Delta (**C**) variants. Scatter plots show lines at the median with interquartile ranges. The dashed lines represent the cut-off value for the test (20 VNT_100_). One-way ANOVA and the nonparametric Kruskal–Wallis test were used to compare the study groups. Asterisks denote statistical significance between the groups (*p < 0.05; ***p < 0.001; ****p < 0.0001). Bar graphs represent the percentage of responders to each variant (**D**).

To investigate whether CoronaVac was able to induce antibodies capable of neutralizing different SARS-CoV-2 lineages, a virus neutralization test was performed through the reduction of the cytopathic effect (VNT100) using serum from previously infected and/or vaccinated individuals against three different SARS-CoV-2 lineages: Brazilian SARS-CoV-2 lineage B reference isolate and Gamma and Delta variants (Fig. 2).

Serum from the VAC group tested against the reference strain made up 70% of the samples that had positive VNT values, with a median VNT100 of 20 (IQ 0–40). Concerning the Gamma and Delta variants we observed a reduction in the proportion of positive samples, presenting approximately 41% and 34%, respectively. The VNT100 values against the variants were also reduced; however, because many of the samples presented values close to the test detection limit, null median VNT values were obtained, impairing this analysis.

Notably, similar to observations from serological analysis, REC/VAC group volunteers showed higher percentages of responder individuals compared to the VAC group (****p < 0.0001), presenting 95% of positive samples for reference strain, and only a slight reduction for Gamma and Delta variants, at 90 and 71% of positive samples, respectively. The median VNT100 value was 160 (IQ 40–320) for the ancestral strain, presenting a neutralizing activity reduction of approximately 2 times for the Gamma variant (median VNT100 of 80, IQ 40–80) and 4 times for the Delta variant (median VNT100 of 40, IQ 0–160).

### Salivary levels of IgA and IgG anti-SARS-CoV-2 antibodies

**Figure 3:**
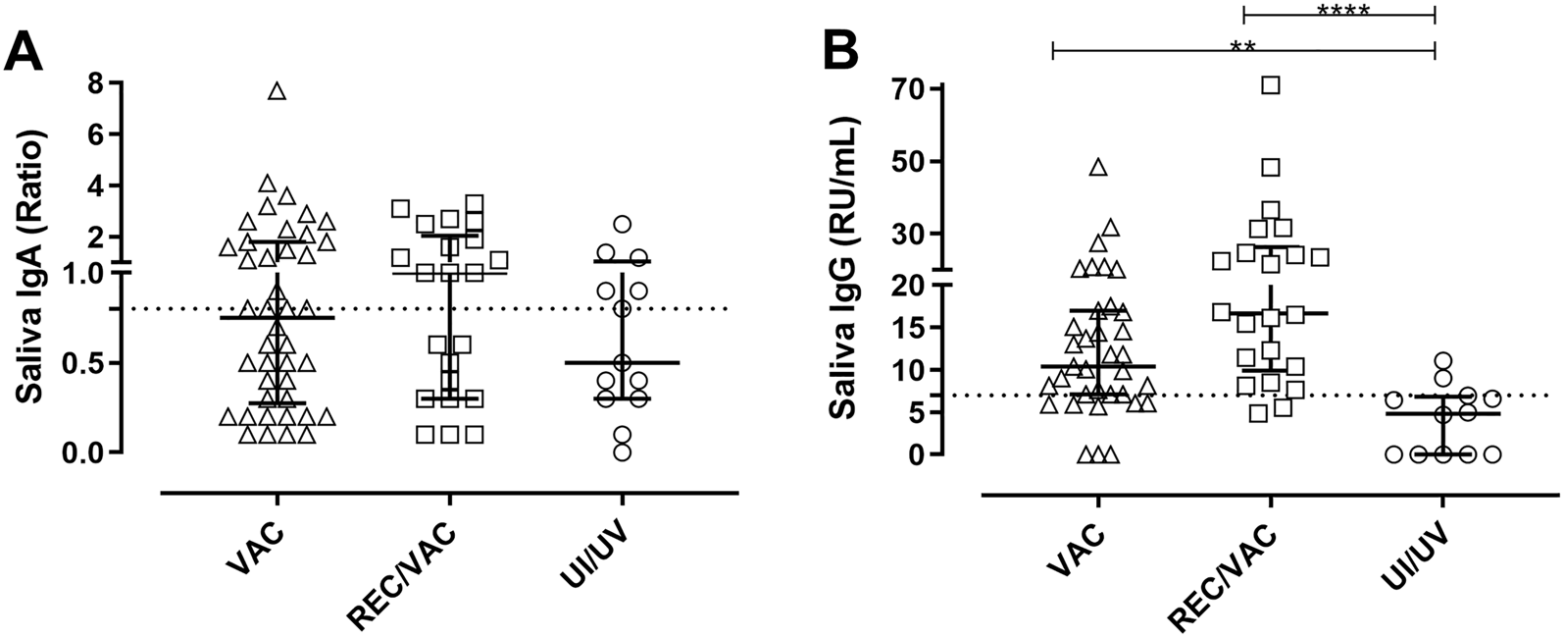
Salivary antibodies against SARS-CoV-2 spike protein. Saliva samples from COVID-19 vaccinated (VAC) (triangles), COVID-19 recovered vaccinated (REC/VAC) (squares) and negative control (UI/UV) individuals (circles) were analysed to determine the IgA (**A**) VAC (n = 42), REC/VAC (n = 22) and UI/UV (n = 13) and to determine the IgG (**B**) VAC (n = 35), REC/VAC (n = 22) and UI/UV (n = 13) antibodies against SARS-CoV-2 spike protein. Scatter plots show lines at the median with interquartile ranges. The dashed lines represent the cut-off value of 0.7 for IgA and 7 RU/mL for IgG. One-way ANOVA and the nonparametric Kruskal–Wallis test were used to compare the study groups. Asterisks denote statistically significant differences between the groups (**p < 0.01; ****p < 0.0001).

The saliva samples were analysed for the presence of IgA and IgG antibodies directed to the S1 (including RBD) of SARS-CoV-2 spike protein (Fig. 3). A total of 77 samples were analysed for IgA.

We were able to detect saliva IgA in 48% of the VAC group and 55% of the REC/VAC group samples; the UI/UV negative control group presented 38% positive samples. No significant differences were found between these groups.

For saliva IgG detection, 70 samples were analysed. The production of saliva IgG antibodies directed to S1 was observed in 77% of VAC, 95% of REC/VAC and 17% of UI/UV group samples. Both the VAC and REC/VAC groups presented IgG production at similar levels but it was significantly higher than that in the UI/UV group. In constrat to the IgA analysis, salivary IgG showed better specificity than salivary IgA, because in the salivary IgG we did not detect a response in most samples from the negative control group.

### IFN-γ production by T-lymphocytes stimulated with SARS-CoV-2 peptides

**Figure 4:**
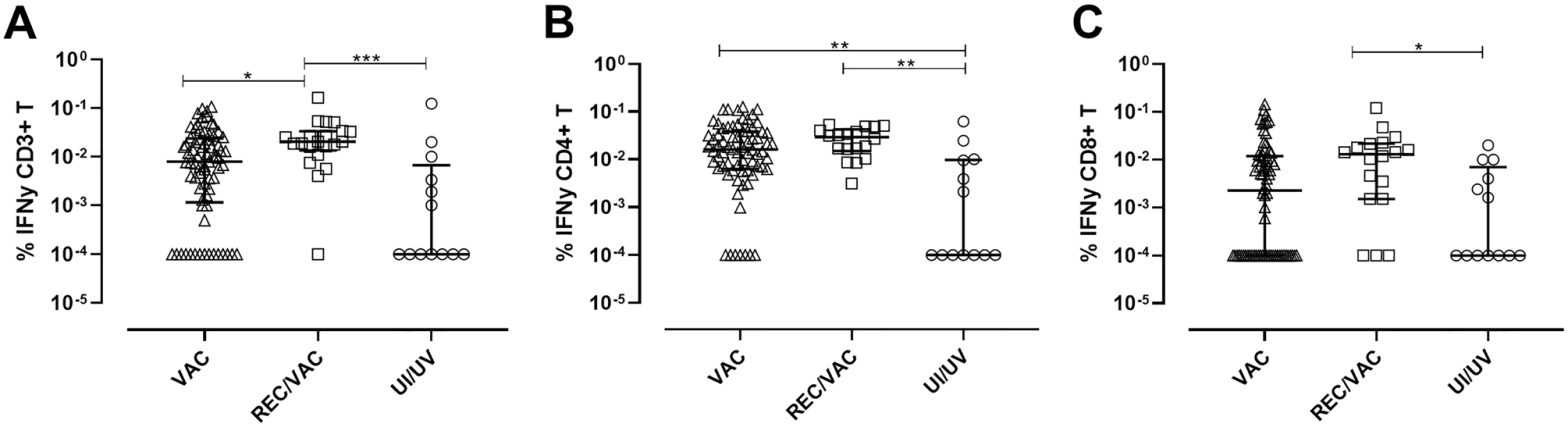
IFN-gamma production by T cells stimulated with SARS-CoV-2 pooled OPPs. PBMCs from vaccinated individuals (VAC) (triangles) (n = 72); COVID-19 recovered vaccinated individuals (REC/VAC) (squares) (n = 21) and uninfected/unvaccinated donors (UI/UV) (circles) (n = 13) were incubated for 18 h with a mixture of grouped SARS-CoV-2 peptide pools (M + N + S) at a final concentration of 1 μg/mL. The logarithmic scale represents the percentage of T cells producing IFN-γ. Scatter plots show lines at the median with interquartile ranges. IFN-γ expression by total lymphocytes (CD3+ T cells) (**A**); CD4+ T (**B**) and CD8+ T-lymphocytes (**C**) was analysed by intracellular staining. One-way ANOVA and the nonparametric Kruskal–Wallis test were used to compare the study groups. Asterisks denote statistically significant differences between the groups (*p < 0.05; **p < 0.01; ***p < 0.001).

The cellular response was analysed through the intracellular detection of IFN-γ in T-lymphocytes stimulated with the SARS-CoV-2 pooled OPPs. In evaluating the total T-lymphocyte population (CD3+), we observed reactive anti-SARS-CoV-2T-cells in 79% of VAC, 100% of REC/VAC, and 46% of UI/UV group samples. The REC/VAC group presented a significantly higher response than the VAC and UI/UV groups (Fig. 4A).

In analysing the T-lymphocyte subpopulations, we observed that the difference in the response level of the VAC and REC/VAC samples was maintained concerning the UI/UV group for the CD4 subpopulation (Fig. 4B), and for the CD8 subpopulation, only samples from the REC/VAC group presented differences concerning UI/UV (Fig. 4C).

In general, SARS-CoV-2 pooled OPPs seem to stimulate a specific response targeted to CD4+ T-lymphocytes. Lastly, lymphocytes from all the donors were able to respond to the positive control (PMA-ionomycin) (Supplementary Fig. S1).

### Integrated data representation

**Figure 5:**
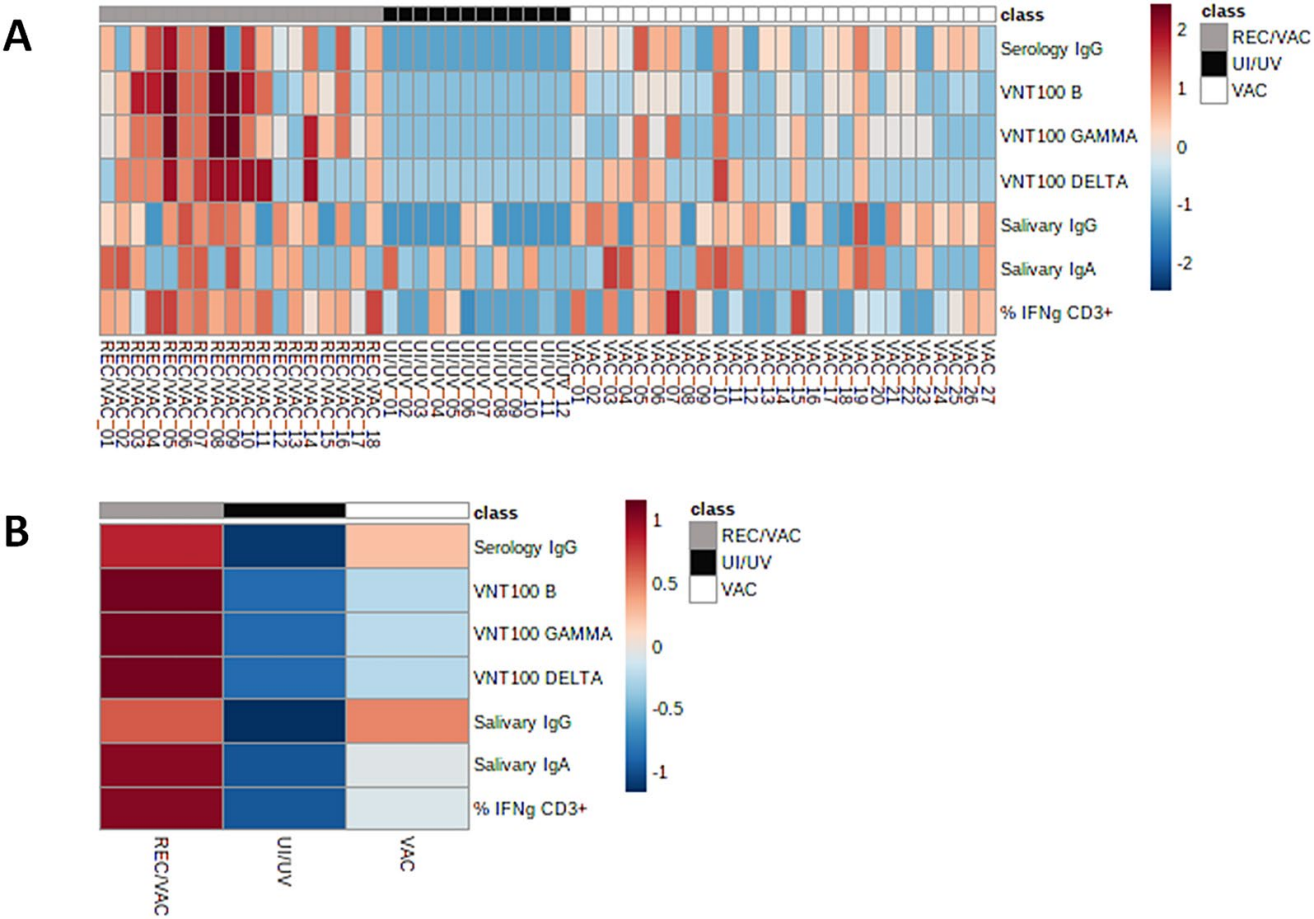
Integrated data representation of analysed samples. (**A**) Hierarchical clustering heat maps without a reorganization of samples and features based on the values regarding the serology IgG, Virus Neutralization Titre (VNT_100_) B, VNT_100_ Gamma, VNT_100_ Delta, salivary IgG, salivary IgA and %IFNg CD3+ for a total of 57 individuals: 27 in the VAC group (COVID-19 vaccinated), 18 in the REC/VAC group (COVID-19 recovered vaccinated individuals), and 12 in the UI/UV group (negative control)). (**A**) The values are shown as rectangles containing different colours corresponding to the levels indicated by the scale bar on the right. Each column represents each individual, and each line represents each variable (aerology IgG, VNT_100_ B, VNT_100_ Gamma, VNT_100_ Delta, salivary IgG, salivary IgA and %IFNγ CD3+). The colours on the top represent each block of the three different groups (VAC—grey, REC/VAC—black and UI/UV—white). (**B**) Heat map based on the group averages. The average values are shown as rectangles containing different colours corresponding to the levels indicated by the scale bar on the right. Each line represents each variable (serology IgG, VNT_100_ B, VNT_100_ Gamma, VNT_100_ Delta, salivary IgG, salivary IgA and %IFNγ CD3+), and each column represents each group. The colours on the top represent each block of the three different groups (VAC—grey, REC/VAC—black and UI/UV—white). The variables were normalized to a 0–100 scale by subtracting the minimum and dividing by the maximum of all the observations. The minimum and maximum values observed here were considered for each variable; a = 0 and b = 100. Second, all the normalized data were log-transformed (base 10). Then, the data were submitted to the Metaboanalyst 5.0 platform.

A total of 57 matched samples (27 from the VAC, 18 from the REC/VAC and 12 from the UI/UV groups) were analysed for the serum IgG antibody quantification, virus neutralizing activity, salivary IgA and IgG and IFN-γ-producing T cells, all of them specific for SARS-CoV-2 antigens. In evaluating these data in an integrated way, two heat maps were generated (Fig. 5). The first heat map (Fig. 5A) represents the values for each volunteer in all the variables, and the second heat map represents the group averages (Fig. 5B).

By analysing individualized data (Fig. 5A), we observed that for the VAC group, positivity for the presence of serum IgG antibodies was not necessarily reflected in serum neutralizing activity, since not all samples that were positive for IgG showed neutralizing activity for the B ancestral lineage nor for the Gamma and Delta variants. In fact, we found no correlation between these variables (data not shown).

On the other hand, particularly for the REC/VAC group, we observed that in general, presence of serum IgG and the virus neutralizing activity responses to the to the B lineage and variants occur in a paired way, with samples presenting simultaneous responses for two or more viral lineages. Reinforcing these data, correlation analysis showed significance between data from serum IgG and all three SARS-CoV-2 lineages (Supplementary Fig. S2A–C) and between the B ancestral, Gamma and Delta variants (Supplementary Fig. S2D–F).

Additionally, a comparison between serum and salivary antibodies showed a distinct profile depending on the group. For serum IgG, the REC/VAC group presented a more intense response than the VAC group, while salivary IgG showed a similar profile between the REC/VAC and VAC groups.

Overall, we observed that the REC/VAC group presented higher responses regarding both the intensity and number of responses for each variable investigated, compared to the VAC group. As expected, the negative control group UI/UV presented the lowest responses (Fig. 5B).

## Discussion

We investigated the anti-COVID-19 post-vaccine response in terms of serum and salivary antibodies, serum virus neutralizing activity and the cellular immune response in matched samples of health care workers who were immunized with two doses of the CoronaVac vaccine who have had (REC/VAC) or did not have (VAC) previous COVID-19.

Serum antibodies were quantified using an assay to recognize IgG antibodies anti-trimeric spike glycoprotein of SARS-CoV-2, which improves the detection of a broader repertoire of neutralizing antibodies. Importantly, since there is now evidence of neutralizing antibodies as a protective correlate for COVID-19 vaccines^18^, we also performed assays to evaluate the ability of these serum samples to neutralize viral activity directly.

Our results showed that CoronaVac immunization was able to induce the production of anti-trimeric spike antibodies in most vaccinated donors. Likewise, in individuals who were previously infected with SARS-CoV-2, vaccination boosted the immune response, significantly increasing antibody levels. These data were reinforced by the results of the virus neutralization test, whose percentage of positivity, although slightly reduced compared to antibody quantification, showed a similar response profile in the assays against the reference B strain, with higher VNT values and percentages of positive responses in the REC/VAC group than in the VAC group.

Muena et al. ^19^ observed that the antibody neutralizing activity was significantly boosted in seropositive individuals after two doses of the CoronaVac or BNT162b2 vaccines, regardless of the time elapsed since COVID-19 symptoms onset. Interestingly, this boost induced by vaccination in COVID-19-recovered individuals is related to the stimulation of B-cell clones. Since these cells retain a large viral repertoire from infection over time, a notable expansion of broad antibodies after vaccination was observed, even with vaccines only based on spike protein, as in mRNA-based vaccines^20^.

The emergence of SARS-CoV-2 variants have raised significant concern, since potentially more infectious and transmissible variants may emerge harboring mutations in the major targeting of neutralizing antibodies, the viral S protein, that can compromise the effectiveness of vaccination programs^4^. Considering that the first generation of vaccines was developed based on the ancestral strain, which did not have mutations, it is relevant to evaluate the neutralizing antibodies of recovered and/or vaccinated individuals against these variants. In this context, the samples were also evaluated for neutralizing activity against two SARS-CoV-2 variants, Gamma and Delta, in addition to the B reference strain.

We observed a reduction in both VNT values and the proportion of samples presenting positive VNT values against the variants, compared to the reference strain. Additionally, these reductions were more pronounced in samples from the VAC group than in the REC/VAC group. These data suggest that although infection- and vaccine-induced immunity can be at least partially retained, Gamma and Delta variants could escape neutralization by antibodies stimulated by the vaccination. Additionally, in previously infected individuals, a boost induced by vaccination seems to be capable of reducing this escape.

A reduction in the potency of neutralizing ability against SARS-CoV-2 variants by antibodies elicited by natural infection or vaccination has been reported. Through a systematic review of data and pooled analysis, Chen et al. ^17^ found that Beta, Gamma and Delta strains significantly escape neutralization mediated by natural infection, while neutralizing titres against the Alpha variant are slightly decreased.

However, for antibody neutralization induced by vaccination, it seems that the immunity induced against variants could depend on the vaccine platform. For mRNA-based vaccine platforms, the Beta variant had the most reduced sensitivity and Alpha, Gamma and Delta showed an intermediate phenotype^21^, while for viral vector-based vaccine platforms, reduced protection against Beta and Delta variants was observed^22^.

Particularly for virus inactivated-based immunization, the Gamma variant can escape from neutralizing antibodies, even after two vaccine doses of CoronaVac, presenting a reduction of approximately 3 times compared to the ancestral strain^23^. In fact, another study comparing the performance of serum derived from CoronaVac vaccinated individuals against the Alpha and Gamma variants showed a reduction of approximately 4.1 times for Alpha and 7.5 times for Gamma variants, compared to the ancestral strain and the decrease in the neutralization titre in relation to the Alpha variant was 1.8 times for Gamma^24^.

CoronaVac was also capable of inducing the production of salivary IgG and, to a lesser extent, salivary IgA directed to the SARS-CoV-2 spike protein. While the detection of salivary IgG was specific, IgA tests showed a low specificity. In addition, a low sensitivity in detecting salivary IgA in COVID-19 patients was reported by Isho et al. ^25^, who observed a sensitivity assay of approximately 50, while IgG test sensitivity was greater than 80%.

Guerrieri et al. ^26^, using the same technique, and Varadhachary et al. ^27^, using a different approach, also showed a large variation in the salivary IgA titre in pre-vaccinated and pre-COVID-19 saliva samples, although they found significantly lower levels compared to those observed in COVID-19 vaccinated and COVID-19 PCR-confirmed individuals, respectively. In this regard, Tsukinoki et al. ^28^ found SARS-CoV-2 cross-reactive sIgA antibodies to spike protein in the saliva of 46.7% of donors who were PCR- and IgM-negative for COVID-19. These results, as in ours, suggest the presence of polyreactive sIgA which can cross-react with a number of antigens, through its N-linked glycan chains, acting as a natural antibody that promptly neutralizes pathogenic microorganism entry at mucosal surfaces^29^.

It is important to emphasize that in saliva, IgA is the most abundant immunoglobulin, and it is primarily produced in salivary glands by local plasma cells primed at mucosal sites and exported by the polymeric Ig receptor (pIgR), unlike salivary IgG, which is primarily derived from serum by passive diffusion through gingival clefts, although some is locally produced^30^. These different sources of salivary IgG and IgA antibodies may explain the better salivary IgG response to the CoronaVac vaccine compared to IgA because any previous contact with the virus could elicit a response by mucosal B lymphocytes, inducing the production of secretory IgA antibodies without generating apparent clinical symptoms. In addition, there are several potential O-linked sites in the hinge region of SIgA antibodies that protect the IgA hinge region from proteases and the secretory component, with its highly glycosylated nature, also has the ability to bind to antigens non-specifically^31,32^.

Additionally, it is important to consider the kinetics of the production and persistence of saliva antibodies. In infected individuals, the peak of salivary IgA production was approximately two weeks after diagnosis, with a rapid decrease after nine weeks, whereas IgG antibodies showed a peak approximately 8 weeks after diagnosis and remained stable until 10 weeks^33^. Reinforcing these data, Ketas et al. ^34^ showed that individuals who received mRNA-based vaccines presented IgA production two weeks after the second vaccine dose. Our samples were collected approximately eight weeks after vaccination, which might have favoured IgG over IgA detection.

Salivary IgG presented similar levels in individuals vaccinated with and without previous COVID-19, unlike those from serum IgG. These different profiles were evidenced by a heatmap analysis (Fig. 5), in which the difference between salivary and serum IgG profiles in VAC individuals was clear. In addition, previous contact with the virus does not seem to change the IgG salivary response. Since saliva IgG is primarily derived from serum by passive diffusion^30^, it would be expected that the saliva reflects the serum content. In this context, our results suggest that CoronaVac also induced the production of IgG antibodies by local immune cells. Corroborating this fact, the analysis between serum and salivary IgG antibody levels from both VAC and REC/VAC showed no correlation between these data (Supplementary Fig. S2G,H), reinforcing that after vaccination, salivary IgG does not correspond to the serum profile. Consistent with these data, the authors have found only a moderate correlation with paired and saliva antibody titres in samples from mRNA-based vaccines recipients^34,35^.

The mucosa and draining lymph nodes of the oropharyngeal tract are one of the entry routes of SARS-CoV-2 in the body, representing an important site for the anti-SARS-CoV-2 immune response initiation^25^. Thus, the presence of specific antibodies in mucosa might prevent or limit virus access through this route^34^, emphasizing the importance of investigating the response in this immune compartment. Although our results cannot be extrapolated to represent protection at this potential site of infection, these findings encourage further investigation about the effect of the vaccine in mucosa sites. To the best of our knowledge, this is the first description of salivary evaluation after immunization with a virus-based vaccine.

An important issue when analysing the immune response after natural infection or vaccination is the durability of the anti-SARS-CoV-2 immune response, which is necessary to promote protective immunity. Memory T and B cells are crucial for long-term protection, particularly specific CD4+ T cells, which elicit a potent B-cell response for antibody affinity maturation^36^. In fact, a decline in circulating antibody titres has been observed a few months after infection or vaccination^12–14^. However, sustained T-cell immunity is reported even in the absence of antibodies^15,16^, suggesting that the cellular response also plays an important role in protective immunity.

The cellular response to inactivated virus-based vaccines has been evaluated in few studies. CoronaVac was able to induce robust circulating and memory B-cell and T-cell responses approximately ten weeks after the second dose^37^. In addition, CoronaVac was able to induce a predominant CD4+ T-cell immune response polarized towards a Th1 profile after stimulation with a mega pool of specific peptides^38^. We were also able to find a greater number of CD4+ T-cells producing IFN-γ than CD8+ T-cells. This observation can be attributed to the OPPs used in our assay, the 15-mer length of which favours the antigenic presentation through MHC class II for CD4+ T cells activation.

Concomitantly, we found higher levels of cellular response in vaccinated individuals with previous SARS-CoV-2 infection compared to COVID-19-naïve vaccinated individuals, showing that cellular response, as well as humoral response, is also boosted by vaccination in previously infected individuals. Similar results were verified in cohorts immunized with mRNA-based vaccines. Individuals with prior infection showed enhanced T-cell immunity after only one^39^ or two doses^40^ of the Pfizer/BioNTech vaccine compared to individuals immunized without previous exposure.

It is important to consider that IFN-γ T-cell production is not necessarily related to protection against infection. Furthermore, the durability of immunity due to vaccination, as well as vaccination after natural infection, requires monitoring over time^41^.

To analyze all the data in an integrated way, heatmaps were generated to elucidate some differences and similarities among the groups (VAC, REC/VAC, and UI/UV) and variables (serum IgG, salivary IgA and IgG, and IFN-γ CD3+). The data generated there showed that the REC/VAC group showed more intense positive responses, for a greater number of variables compared to the VAC group, suggesting that vaccination may have worked as a booster, reinforcing the anti-SARS-CoV-2 immune response.

Regarding the UI/UV group, we observed that some individuals had salivary IgA production specific for SARS-CoV-2 and IFN-γ production in response to stimulation with specific peptides, although they did not present a positive PCR test, positive IgG serology, or reported symptoms of COVID-19. In addition to the possibility of low test specificity, a possible explanation would be that the presence of IgA or T-cell response could reflect a viral exposure that did not result in systemic infection but was sufficient to trigger a mucosal or cellular memory response^34,42^.

Our study has several limitations. First, the study population was mostly composed of female individuals. Second, the negative control group (UI/UV) was composed of younger volunteers compared to the VAC and REC/VAC groups. Third, the saliva collection time for IgA analysis seems not to have been ideal for peak response analysis. Fourth, analyses of some immune components could not be performed, either due to the lack of samples or assay limitations, which did not allow for integrated analyses using all the samples. Lastly, our study lacked a control group composed of unvaccinated recovered COVID-19 individuals, which could clarify the role of the CoronaVac vaccine in the profile of salivary IgG production.

In conclusion, our study provides evidence that the CoronaVac vaccine was able to induce serum and saliva IgG directed to SARS-CoV-2 spike protein, serum neutralizing activity against the reference SARS-CoV-2 strain and IFN-γ production by T cells stimulated with specific peptides. Additionally, previously COVID-19 infected individuals presented an increased response for all variables investigated compared to vaccinated uninfected individuals. To the best of our knowledge, this is the first description of the immune profile, including neutralizing activity against SARS-CoV-2 variants, in previously infected individuals immunized with a virus inactivated-based vaccine.

## Methods

### Study subjects

A total of 115 volunteers were included in this study. One hundred and two of them are health care workers (HCW) at the Instituto da Crianca (Sao Paulo, Brazil) who were immunized during an institutional anti-COVID-19 vaccination campaign. Twenty-two of these HCWs had previous SARS-CoV-2 infections, as confirmed by PCR or serology antibody test, and they were analysed as a separate group (REC/VAC), and eighty of them had no previous history of SARS-CoV-2 infection (VAC). Samples were collected between 47 and 85 days after the 2nd dose of the CoronaVac vaccine. The negative control group consisted of 13 individual non-employees, who were recruited from among our laboratory students, with participants from the UI/UV group were invited to fill out a pre-screening questionnaire used to discard previous infection or exposure to SARS-CoV-2. Moreover, a rapid test for the detection of anti-SARS CoV-2 IgM antibody (KHB diagnostic kit, Shanghai Kehua Bio-Engineering Co., Ltd.) was performed to detect active infection qualitatively. Samples positive for IgM were not included in the analysis. A summary of the groups studied, samples collected and tests performed are represented in Supplementary Fig. S3.

Written informed consent was obtained according to the protocols of the Hospital das Clinicas Ethical Committee (CAPPesq) (Sao Paulo, Brazil) under approval protocol #4.360.357. All the participants gave informed consent at the time of recruitment for the study.

### Sample collection

Heparinized blood samples were collected to obtain peripheral blood mononuclear cells (PBMCs) for cell culture and serum samples for a serological analysis of IgG antibodies.

Unstimulated whole saliva samples were collected 1 h after oral rinsing, using sterile vials which were kept on ice. They were then immediately centrifuged at 15,000 rpm for five minutes to ensure that the saliva samples were clear, followed by storage at − 80 °C until use.

### Quantitative assay for serum IgG antibody detection

An automated indirect chemiluminescence immunoassay was performed for the quantitative determination of IgG antibodies, including neutralizing antibodies against the trimeric spike protein of SARS-CoV-2 in serum samples, using the LIAISON^®^ SARS-CoV-2 TrimericS IgG assay (DiaSorin S.p.A., Saluggia, Italy). The Trimeric protein is a stabilized native form of the spike that improves the detection of neutralizing antibodies.

### Cytopathic effect-based virus neutralization test (CPE-VNT)

The CPE-VNT was carried out with SARS-CoV-2 variant B (MT350282), Variant Gama (EPI_ISL_1060981) and variant Delta (EPI_ISL_2965577) in 96-well plates containing 5 × 10^4^cells/mL of Vero cells (ATCC CCL-81).

The plasma samples were initially inactivated for 30 min at 56 °C. We used 8 dilutions (twofold) of each plasma (1:20 to 1:2560) mixed vol/vol with the virus (100 TCID50 per well) and pre-incubated at 37 °C for 1 h to allow virus neutralization. Then the mixture (plasma + virus) was transferred onto the confluent cell monolayer and incubated for 3 days at 37 °C and under 5% CO2. After 72 h, the plates were analyzed directly with transmitted-light bright-field microscopy (Olympus Co., Tokyo, Japan). Gross cytopathic effect can be observed on Vero cells, after 72 h, being able to distinguish the presence/absence of viral cytopathic effect caused by SARS-CoV-2.

Virus neutralization titer referred to as VNT_100_ is described as the highest dilution of serum that neutralized virus growth. For double check of the titers, the plates were fixed and stained for 30 min with 0.2% Naphthol blue black solution (Sigma-Aldrich Co., Deisenhofen, Germany) and then photographed for documentation of culture morphology. In each assay, a strong, assured internal positive control serum (RT-qPCR positive + PRNT90 > 640)^43^ was used, as a negative pre-outbreak serum sample.

The method described here was adapted from Nurtop et al. ^44^ and has been widely used for SARS-CoV serological studies^45–50^. All the procedures related to CPE-VNT were performed in a biosafety level 3 laboratory, following WHO recommendations^51^.

### Detection of anti-S1 IgG and IgA salivary antibodies through ELISA

A semiquantitative determination of salivary IgA antibodies against the S1 domain of the spike protein (including RBD) was performed by using a Euroimmun Anti-SARS-CoV-2 S1 ELISA IgA kit (cat. #EI 2606-9601A) after optimization for saliva, in accordance with the manufacturer’s protocol. After a dilution correction (1:50), the results were presented as a ratio calculated by the optical density value of the samples over the optical density value of the calibrator (both read at 450 nm, using a reference wavelength of 620 nm). Ratios < 0.8 were considered negative, undetermined for all values between 0.8 and 1.1 and positive > 1.1, according to the manufacturer’s instructions.

A quantitative determination of salivary IgG directed to the S1 domain was performed using the anti-SARS-CoV-2 QuantiVac ELISA IgG (cat. #EI 2606-9601-10G) according to the manufacturer’s instructions. For salivary IgG, after dilution correction (1:100), the IgG concentrations were determined using the reference standard concentrations to construct the 6-point calibration curve. The results were reported as RU/mL, considering IgG levels above 7 RU/mL as positive.

### T-cell responses to SARS-CoV-2 antigens

Overlapping peptide pools (OPPs) (15-mers with 11 amino acid overlaps) covering the immunodominant sequence domain of representing spike (S) (PepTivator^®^ SARS-CoV-2 Prot_S; #130-126-700), the complete sequence of the membrane (M) (PepTivator^®^ SARS-CoV-2 Prot_M; #130-126-702) and nucleocapsid (N) (PepTivator^®^ SARS-CoV-2 Prot_N; #130-126-698) SARS-CoV-2 proteins (Miltenyi Biotec, CA, USA) were used to stimulate the T-cell response as previously described^52^.

The cells were stained with an amine-reactive fixable live*/*dead stain (Gibco; Life Technologies), anti-CD3 PE-Cy5 antibody (clone 7D6, Invitrogen; Thermo Fisher Scientific), CD4 PE antibody (clone RPA-T4, BD Biosciences, CA, USA), anti-CD8 APC-H7 antibody (clone SK1, BD Biosciences, CA, USA) and intracellular marker interferon-gamma (IFN-γ) V450 (clone B27, BD Biosciences, CA, USA) using a Cytofix/Cytoperm kit (BD Bioscience) as recommended by the manufacturer. PBMCs were acquired on an LSR Fortessa (BD Bioscience), and the analysis, as exemplified in Supplementary Fig. S4, was performed using FlowJo v. 10.6.1 software (Ashland, OR: Becton, Dickinson and Company).

### Statistical analysis

One-way ANOVA and nonparametric Kruskal–Wallis tests were used to compare the three study groups. To compare variables related to the vaccinated groups, Student’s t tests with nonparametric Mann–Whitney tests were performed. Correlations among variables were established using Spearman’s correlation (r2 > 0.7 and *p* < 0.05). Graphical representations were created using GraphPad Prism v. 8 software (GraphPad Software). *p* < 0.05 was considered statistically significant.

Two hierarchical clustering heat maps without the reorganization of samples and features based on the values regarding the serology IgG, Virus Neutralization Titre (VNT_100_) B, and VNT_100_ Gamma, VNT_100_ Delta, salivary IgG, salivary IgA and % IFN-γ CD3+ cells were designed using the MetaboAnalyst 5.0 platform (https://www.metaboanalyst.ca). Data normalization methods were used to make the variables, which were measured in different scales, have comparable values. First, the variables were normalized to bring the data to the 0 to 100 scale by subtracting the minimum and dividing by the maximum of all the observations (formula shown below). The minimum and maximum values observed here were considered for each variable; a = 0 and b = 100. Second, the normalized data were log-transformed (base 10). Then, the data were analysed on Metaboanalyst 5.0, and the features were subjected to Autoscale (which were mean-centred and divided by the standard deviation of each variable). For this analysis, we considered only samples from volunteers who were evaluated through all tests performed, for a total of 57 samples: 27 samples from the VAC group, 18 for the REC/VAC group and 12 for the UI/UV group, and the second heat map was generated using the group averages.

$$Transformed\, value=a+\frac{\left(Value-Minimum\right)(b-a)}{Maximum-Minimum}.$$

## Supplementary Information

Supplementary Figures.
