# Supplementary material for: Salivary, serological, and cellular immune response to the CoronaVac vaccine in health care workers with or without previous COVID-19
Source: Sci Rep. 2022 Jun 16;12:10125. doi: 10.1038/s41598-022-14283-x (PMC9202665; doi:10.1038/s41598-022-14283-x)
Supplement: Supplementary file 1 — Supplementary Figures. [file 41598_2022_14283_MOESM1_ESM.docx]

**SUPPLEMENTARY FIGURE LEGENDS**

**Figure S1.** **T-cell responses to PMA/ionomycin.** PBMCs from vaccinated individuals (VAC) (triangles) (n = 72); COVID-19 recovered vaccinated individuals (REC/VAC) (squares) (n = 21) and uninfected/unvaccinated donors (UI/UV) (circles) (n = 13) were incubated for the last 6 h with PMA (30 ng/mL) and ionomycin (0.3 mg/mL) (positive control). The logarithmic scale represents the percentage of CD3+ (A), CD4+ (B) and CD8+ (C) T-cells producing IFN-γ. Scatterplots show lines at the median with interquartile ranges.

**Figure S2.** **Correlation analysis.** Serum samples from recovered COVID-19 vaccinated individuals (REC/VAC) (n = 21) were analysed for correlation between IgG antibodies against S1 of the SARS-CoV-2 spike protein and virus neutralization activity (VNT) against SARS-CoV-2 reference lineage B (A), Gamma (B) and Delta (C) variants. Data from VNT assays of the REC/VAC group were analysed for correlation between reference lineage B and Gamma variant (D); reference lineage B and Delta variant (E) and Gamma and Delta variants (F). Samples from vaccinated individuals (VAC) (triangles) (n = 35) (G) and recovered COVID-19 vaccinated individuals (REC/VAC) (squares) (n = 22) (H) groups were analysed for correlation between IgG antibodies directed to S1 (including RBD) of the SARS-CoV-2 spike protein in serum and saliva. All correlations were established using Spearman’s correlation (r2 > 0.7 and p < 0.05).

**Figure S3.** **Brief methodology representation**. The study subjects are represented in the following groups: vaccinated health care workers with previous COVID-19 (REC/VAC) (teal); vaccinated health care workers without previous COVID-19 (VAC) (orange); and uninfected and unvaccinated donors (UI/UV) as negative controls (pink). Samples of saliva, heparinized blood, and serum were collected from all subjects. Variable analyses included the determination of IgA and IgG in saliva by ELISA against the S1 domain of the SARS-CoV-2 spike protein; IgG measurement in serum by indirect chemiluminescence against the SARS-CoV-2 trimeric spike protein; IFN-γ T lymphocyte production by PBMCs stimulated with SARS-CoV-2 peptide pools, and the neutralizing activity of serum antibodies against reference strain B, Gamma and Delta SARS-CoV-2 variants were evaluated using a virus neutralization test.

**Figure S4.** Representative flow cytometry plots of IFN-γ production by T cells from one REC/VAC individual. The SSC-A x time analysis strategy was employed to exclude possible unstable cell flow (A); after that, doublet exclusion was performed (B). The size and granularity patterns of the cells were analysed by SSC and FSC (C); therefore, within the live cell population (D), CD3+ T cells were analysed (E) for IFN-γ production in the negative control (without stimulation) (F) or following stimulation with SARS-CoV-2-overlapping peptide pools (OPPs) (G) or phorbol myristate acetate and ionomycin (positive control) (H). The production of IFN-γ by CD3+ CD4+ T cells (I) without stimulation (J) or after stimulation with OPPs that were polled (K) or the positive control (L) was analysed. This same strategy was applied to study IFN-γ production by the CD3+ CD8+ T-cell subtype. Samples were acquired on an LSR Fortessa (BD). Flow cytometry data were analysed using FlowJo v. 10 software.

**SUPPLEMENTARY MATERIAL**

**Figure S1**


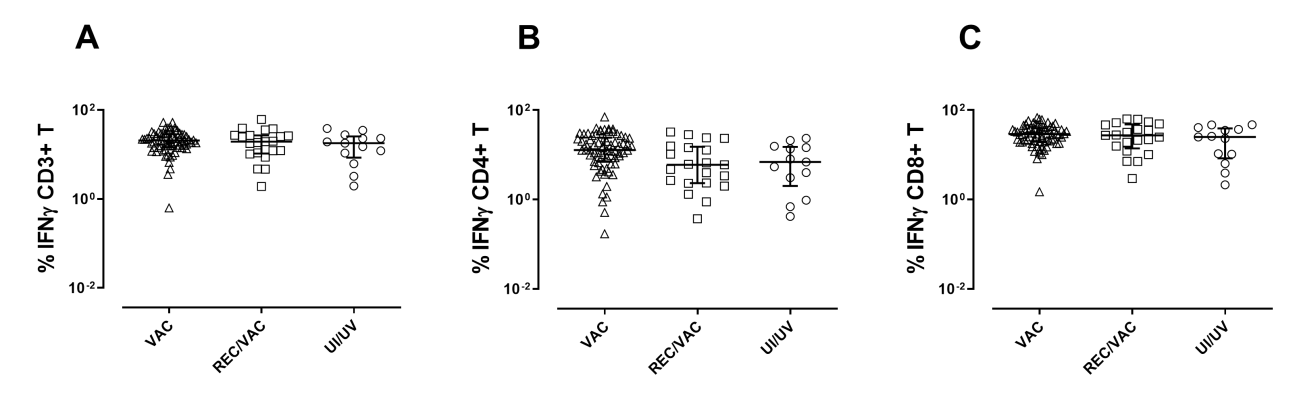


**Figure S2**

**
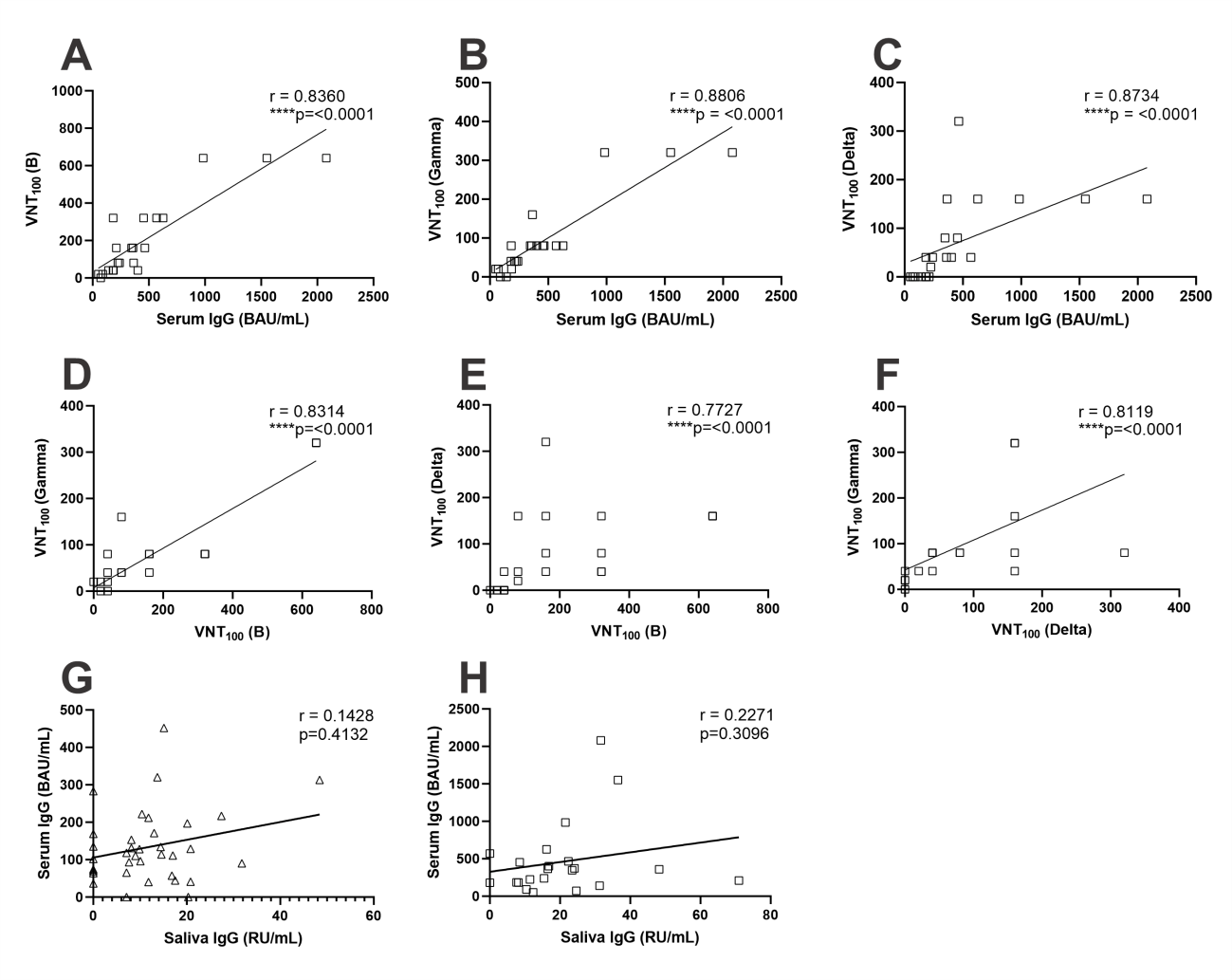
**

**Figure S3**

**
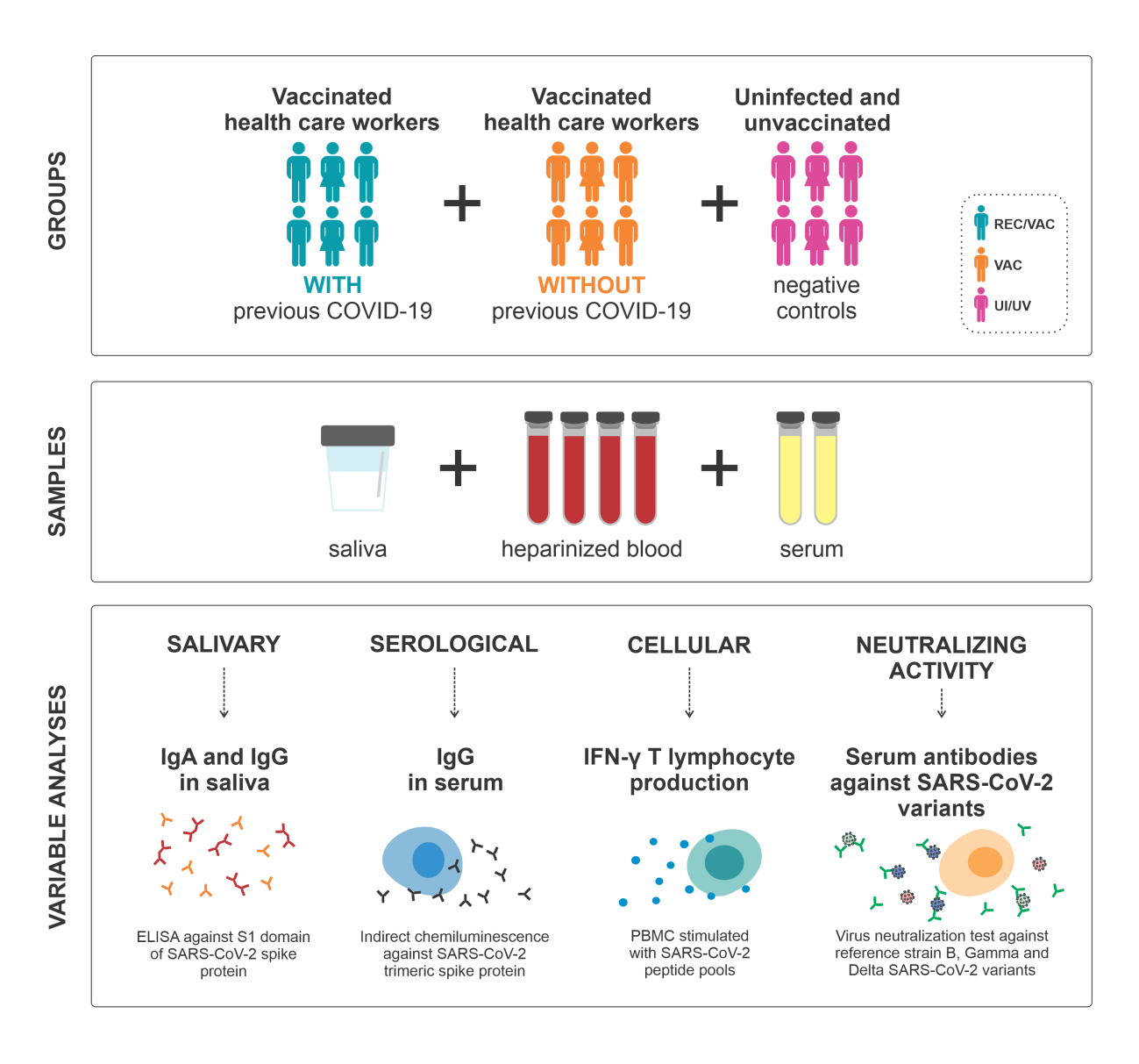
**

**Figure S4**

**
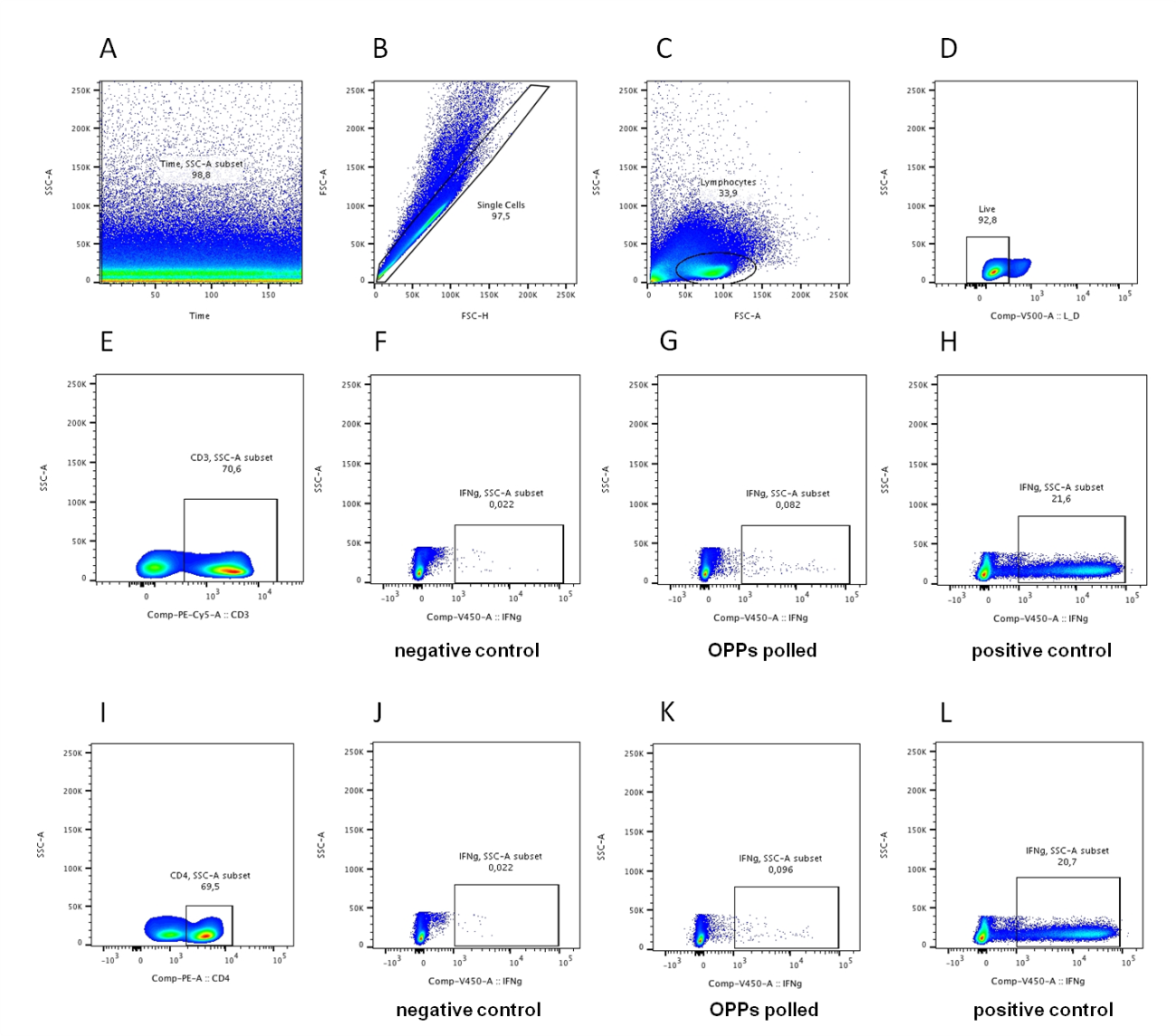
**
